# Enhancing Metabolism and Milk Production Performance in Periparturient Dairy Cattle through Rumen-Protected Methionine and Choline Supplementation

**DOI:** 10.3390/metabo13101080

**Published:** 2023-10-14

**Authors:** Bingjian Huang, Muhammad Zahoor Khan, Xiyan Kou, Yinghui Chen, Huili Liang, Qudrat Ullah, Nadar Khan, Adnan Khan, Wenqiong Chai, Changfa Wang

**Affiliations:** 1 Liaocheng Research Institute of Donkey High-Efficiency Breeding and Ecological Feeding, Liaocheng University, Liaocheng 252000, China2210150218@stu.lcu.edu.cn (Y.C.);; 2 College of Life Sciences, Liaocheng University, Liaocheng 252059, China; 3 Faculty of Veterinary and Animal Sciences, University of Agriculture, Dera Ismail Khan 29220, Pakistan; qudrat.ullah@uad.edu.pk; 4 Livestock and Dairy Development (Research) Department Khyber Pakhtunkhwa, Peshawar 25120, Pakistan; 5 Genome Analysis Laboratory of the Ministry of Agriculture, Agricultural Genomics Institute at Shenzhen, Chinese Academy of Agricultural Sciences, Shenzhen 511464, China

**Keywords:** dairy cattle, periparturient period, RPM, RPC, milk production, metabolism, nitrogen utilization

## Abstract

For dairy cattle to perform well throughout and following lactations, precise dietary control during the periparturient phase is crucial. The primary issues experienced by periparturient dairy cows include issues like decreased dry matter intake (DMI), a negative energy balance, higher levels of non-esterified fatty acids (NEFA), and the ensuing inferior milk output. Dairy cattle have always been fed a diet high in crude protein (CP) to produce the most milk possible. Despite the vital function that dairy cows play in the conversion of dietary CP into milk, a sizeable percentage of nitrogen is inevitably expelled, which raises serious environmental concerns. To reduce nitrogen emissions and their production, lactating dairy cows must receive less CP supplementation. Supplementing dairy cattle with rumen-protected methionine (RPM) and choline (RPC) has proven to be a successful method for improving their ability to use nitrogen, regulate their metabolism, and produce milk. The detrimental effects of low dietary protein consumption on the milk yield, protein yield, and dry matter intake may be mitigated by these nutritional treatments. In metabolic activities like the synthesis of sulfur-containing amino acids and methylation reactions, RPM and RPC are crucial players. Methionine, a limiting amino acid, affects the production of milk protein and the success of lactation in general. According to the existing data in the literature, methionine supplementation has a favorable impact on the pathways that produce milk. Similarly, choline is essential for DNA methylation, cell membrane stability, and lipid metabolism. Furthermore, RPC supplementation during the transition phase improves dry matter intake, postpartum milk yield, and fat-corrected milk (FCM) production. This review provides comprehensive insights into the roles of RPM and RPC in optimizing nitrogen utilization, metabolism, and enhancing milk production performance in periparturient dairy cattle, offering valuable strategies for sustainable dairy farming practices.

## 1. Introduction

Effective nutritional management during the periparturient period plays a crucial role in optimizing lactational performance among lactating dairy cows [1,2,3]. Previous research has emphasized that variations in dry matter intake (DMI) and concentrations of non-esterified fatty acids (NEFA) have a significant impact on both fertility and subsequent milk production outcomes [3,4]. The common practice of implementing a traditional dry period before parturition inadvertently results in reduced DMI, thereby limiting energy intake. Consequently, this leads to a state of negative energy balance, causing a surge in NEFA and β-hydroxybutyric acid (BHBA) levels. Elevated levels of NEFA and BHB can incite compromised immune function, particularly by dampening neutrophil activity in the lead-up to calving [5]. In addition, that elevation of NEFA and BHB regulate oxidative stress, which is followed by increased inflammatory changes and suppressed immunity [2,6]. The suppressed immunity, elevated oxidative stress and inflammatory changes due to negative-energy-balance-causing abnormal levels of NEFA and BHB are the key factors that expose dairy cattle to various diseases including mastitis [7,8], ketosis, retained placenta, and ketosis [9,10]. Furthermore, escalated NEFA and BHB levels with poor health also exert an adverse influence on lactational performance [7].

In the realm of dairy cattle nutrition, the significance of RPM and RPC extends beyond their roles in metabolism and milk production enhancement. These compounds have garnered substantial attention for their contributions to periparturient health disorders in dairy cattle. Research has elucidated the pivotal roles of RPM and RPC in alleviating oxidative stress, mitigating inflammatory changes, and bolstering immunity [2,11]. This body of evidence underscores their potential in promoting overall bovine health. Furthermore, several field experimental trials have consistently reported the clinical implications of RPM and RPC supplementation [12,13]. Notably, RPM has emerged as a crucial factor in controlling and reducing the risk of mastitis in dairy cattle during the periparturient period [2,12,13]. Additionally, Dai et al. [14] demonstrated through in vitro experimental procedures that methionine and arginine treatment effectively alleviated inflammatory changes and oxidative stress induced by lipopolysaccharide (LPS) in bovine mammary epithelial cells (BMECs). Moreover, it is well known that ruminants possess a limited capacity to secrete very low-density lipoproteins (VLDL) from the liver, which can hinder the export of hepatic triacylglycerols, ultimately resulting in hepatic lipidosis. However, the supplementation of 25.8 g/d of RPC has been found to significantly increase hepatic triacylglycerol secretion, thereby reducing the incidence of hepatic lipidosis in dairy cows [15,16]. Other studies have indicated that RPM can reduce the occurrence of subclinical hypocalcemia [17]. However, it should be noted that RPM supplementation did not appear to reduce the incidence of other health disorders, including retained placenta and ketosis, in dairy cattle.

To enhance milk production, dairy cattle during the periparturient period are provided with a high-protein diet. The dairy industry faces a well-established phenomenon where dairy cows convert 20 to 35% of dietary crude protein (CP) into milk, with the remaining nitrogen being excreted in manure, leading to detrimental environmental consequences [18,19,20,21]. In response to this issue, reducing CP supplementation has become a significant focus in the field of lactating ruminants in the United States, as CP serves as the primary nitrogen source [22]. The U.S. dairy sector is under increasing pressure to reduce emissions of nitrogen, phosphorus, and greenhouse gases [23]. Nitrogen, in particular, represents a primary pollutant originating from dairy farm operations, contributing to instances of nitrate contamination in groundwater, the eutrophication of surface water, and emissions of ammonia and nitrous oxide into the atmosphere [24,25].

Implementing the recommended reduction in mobilized protein supplementation, as advocated by the National Research Council (NRC) in 2001 [26], could lead to improved nitrogen utilization for milk production within dairy cattle [27]. However, empirical evidence has shown that dietary protein intake below the recommended threshold may compromise milk production, milk protein yield, and DMI [28,29]. In such cases, the incorporation of RPM [30,31,32,33,34], along with RPC supplementation [35,36], has emerged as an effective strategy to mitigate the adverse consequences resulting from inadequate dietary protein intake.

RPM and RPC have received significant attention due to their notable contributions in promoting milk production performance and optimizing nitrogen utilization within dairy cattle [36,37,38,39,40,41]. A series of studies have consistently confirmed the crucial role of amino acids in orchestrating the regulation of milk and its components in dairy cattle [42,43,44,45,46]. Consequently, a study has emphasized the indispensable role of RPM in milk production and metabolism, as evidenced by in vitro experiments using mammary epithelial cells. These studies highlight the relevance of concentrations of lysine (Lys) and RPM in the medium, not only in optimizing milk protein synthesis but also in influencing amino acid transport and signal transduction pathways that impact the expression of genes associated with milk protein synthesis [47]. Optimal levels of Lys and RPM, along with a supplemental Lys-to-RPM ratio approximating 3:1, have been identified as catalysts for eliciting the expression of genes instrumental in milk protein transcription and translation, ultimately resulting in the peak of casein production and cell proliferation rates [26,48]. RPM, in synergy with other amino acids, has similarly shown positive effects on the production performance and the metabolic aspects of dairy cattle. Importantly, a study has documented that milk yield [12], as well as energy-corrected milk (ECM), milk fat, protein, and lactose percentage levels, exhibited enhancements in cows supplemented with RPM and lysine compared to the control group [49,50]. Furthermore, the study ascertained that supplementation with RPM and RP-lys preserved the post-calving body condition score (BCS), enhanced DMI, and reduced the blood concentrations of BHBA [49,50].

The importance of RPM in milk production is accentuated by its status as the rate-limiting amino acid for lactating dairy cows, especially when fed protein supplements high in lysine but low in methionine, such as blood meal and soybean meal. To mitigate this constraint and augment the post-ruminal methionine supply, the provision of RPM has emerged as an effective strategy. A multitude of studies have outlined the advantages conferred by RPM supplementation, including the economization of dietary protein and the enhancement of overall lactational performance in dairy cows, particularly in diets formulated around standard North American ingredients [51,52]. Recent academic efforts have also focused on RPM supplementation in the diets of transition cows, revealing that achieving a Lys-to-RPM ratio approximating 2.9:1 as a fraction of metabolizable protein results in increased milk yield, partly attributable to increased DMI and the potential for the more effective utilization of bodily lipid reserves [52].

In addition, methionine, as the only amino acid containing sulfur, plays the role of a precursor for other sulfur-containing amino acids, namely cysteine, homocysteine (Hcy), and taurine, all of which play critical roles in various methylation reactions. The metabolic journey of methionine begins with its conversion into S-adenosylmethionine (SAMe), a key cofactor in methionine intermediate metabolism used in methylation reactions. SAMe serves as a methyl donor for amino acid residues within proteins, DNA, RNA, and small molecules, thereby influencing a wide range of biological processes [53]. Furthermore, methionine acts as a precursor for hydrogen sulfide, taurine, and glutathione, all of which have demonstrated their effectiveness in counteracting oxidative stress caused by various oxidants, thus protecting tissues from damage [54]. As a result, the dietary inclusion of rumen-protected methyl donors, such as RPM, has advantages in meeting the needs of cows during the peak of lactation, when the outflow of methylated compounds in milk is increased.

Extensive academic research has highlighted the positive correlation between RPC and improved production performance, while also providing improvement for metabolic challenges faced by dairy ruminants [55]. This suggests that RPC, similar to a vitamin-like substance, plays a crucial role in animal production, reproduction, and overall health [35]. Of particular note, an insightful meta-analysis study has illuminated the effectiveness of RPC, as evidenced by its ability to increase the production of ECM, elevate milk yields, and enrich the composition of milk constituents [56].

Given the significant importance attributed to RPM and RPC, this review aims to comprehensively explore their fundamental roles as indispensable nutritional strategies. The scope of this inquiry is directed towards enhancing metabolic processes, optimizing nitrogen utilization, and ultimately improving lactational performance within the domain of dairy cattle.

## 2. Materials and Methods

We conducted a comprehensive literature review encompassing studies that investigate the influence of RPM and RPC on the metabolic processes, nitrogen utilization, and milk production performance in periparturient dairy cattle. The identification of pertinent articles for this review was accomplished through rigorous search methodologies utilizing reputable academic databases, including PubMed, ScienceDirect, Web of Science, SpringerLink, Scopus, and Google Scholar.

The search strategy was thoroughly designed, employing key terms such as “milk production”, “metabolism”, “one-carbon metabolic pathway”, “amino acid metabolism”, “periparturient dairy cattle”, “RPC”, and “RPM” to retrieve the relevant literature. Our focus was primarily directed towards sourcing data published in the English language and featured in highly regarded peer-reviewed journals. Specifically, we prioritized articles published from the year 2000 onwards to ensure the inclusion of contemporary research findings.

To maintain the highest standards of academic rigor, we deliberately excluded content in the form of conference abstracts, books, book chapters, and unpublished findings from our review. This stringent selection process ensured that the information discussed in this review is grounded in the credible and peer-reviewed scientific literature, contributing to the overall reliability and integrity of our analysis.

## 3. Interplay of Methionine and Choline in One-Carbon Metabolism and Amino Acid Regulation

Methionine plays a crucial role in one-carbon metabolism [57], coordinating a complex series of biochemical processes that transfer one-carbon (methyl) groups in various forms [58,59,60,61,62]. This metabolic pathway is essential for critical cellular functions, including the synthesis of DNA and RNA, amino acid metabolism, and the production of S-adenosylmethionine (SAMe), a universal methyl donor crucial for methylation reactions throughout the body [63,64]. Methionine itself acts as the precursor to SAMe, a key molecule in this pathway, formed by combining methionine with adenosine triphosphate (ATP). SAMe, in turn, donates its methyl group to various substrates, participating in a wide range of biochemical reactions, including DNA and RNA methylation, protein methylation, and the synthesis of neurotransmitters, which are essential for mood regulation and brain function.

As SAMe exhausts its methyl group in methylation reactions [57], it transforms into S-adenosylhomosysteine (AdoHcy) [65]. This conversion involves further steps, including transferring a methyl group to Hcy to regenerate methionine. Methionine also initiates protein synthesis, a fundamental process responsible for constructing all body proteins, from enzymes to structural proteins, as well as those involved in various cellular functions. It actively participates in transmethylation reactions, influencing the synthesis of amino acids, neurotransmitters, DNA, RNA, and lipids. Furthermore, methionine contributes to its own regeneration from Hcy and the production of vital molecules such as creatine, choline, and epinephrine. It also aids in cysteine synthesis through transsulfuration, which is essential for the formation of protein disulfide bonds and the production of the antioxidant glutathione [66,67].

Choline plays a vital role in regenerating methionine from Hcy by generously donating methyl groups in one-carbon metabolism [68]. Additionally, choline is involved in creatine synthesis, which is crucial for muscle energy metabolism, and serves as a precursor for choline-containing amino acids such as betaine [69], and sarcosine, contributing to Hcy conversion into methionine and methyl group detoxification. In conclusion, methionine and choline intricately interconnect in amino acid metabolism [70]. Methionine supports protein synthesis, transmethylation reactions, and cysteine synthesis, while choline contributes to methionine regeneration [71], creatine synthesis, and the production of choline-containing amino acids. Together, these processes ensure the availability of amino acids, crucial for protein production, cellular functions, and overall health in dairy cattle and other organisms.

From the above discussion, it can be concluded that methionine and choline have distinct but interconnected roles in amino acid metabolism. Methionine serves as an essential amino acid required for protein synthesis and participates in transmethylation reactions and cysteine synthesis. Choline, on the other hand, is crucial for regenerating methionine from Hcy, contributing to the one-carbon metabolism pathway, and plays a role in the synthesis of creatine and choline-containing amino acids. These processes collectively ensure the availability of amino acids for protein production, cellular functions, and overall health in dairy cattle and other animals.

## 4. The Role of RPM and RPC in Metabolism and Milk Production Performance of Dairy Cattle

### 4.1. RPM Role in Metabolism and Nitrogen Utilization of Dairy Cattle

Methionine, an indispensable amino acid, plays a crucial role in shaping the physiological development, metabolism, and growth of mammals [72,73,74]. Seymour particularly emphasizes the importance of this amino acid in metabolic processes, especially as a precursor to essential compounds required for vital physiological functions [75]. Methionine serves as a fundamental building block for compounds such as succinyl-CoA, Hcy, cysteine, creatine, and carnitine. Furthermore, its active involvement in the biosynthesis of SAMe is essential for polyamine, creatine, and phosphatidylcholine metabolism [76].

The role of methionine extends far beyond its basic building-block function. It actively participates in cellular methylation reactions and sulfur recycling processes, with the ability to undergo enzymatic conversion to L-methionine sulfoxide [76,77]. The resulting product of this conversion, cysteine, plays a critical role in fundamental cellular functions, including protein translation, glutathione synthesis, and taurine production [78,79]. The consequences of insufficient methionine levels are significant, leading to detrimental effects such as small intestine atrophy, suppressed epithelial growth in neonatal animals, reduced goblet cells, and diminished glutathione content within the small intestine [80].

Within the gastrointestinal tract (GIT), approximately 20% of dietary methionine is absorbed and utilized [81]. Research by Shoveller et al. [82] indicates that neonatal piglets’ parenteral methionine requirement approximates 69% of the enteral requirement. Additionally, methionine catabolism has been found to be more pronounced in extracellular cells outside pig enterocytes, particularly in the portal-drained viscera and intestinal mucosa, as highlighted by Blachier et al. [83]. The dynamic interaction of methionine with choline, as emphasized by Swain and Johri [84], underscores its significance in antibody (IgG) production. Conversely, methionine deficiency leads to a decrease in relative lymphoid organ weight, ultimately impacting overall growth performance [85]. The balance is delicate, as excessive methionine intake has been associated with growth depression [86].

A comprehensive exploration of the role of amino acids in nitrogen management has been a topic of close scrutiny [32,87,88,89,90]. Recent research has provided significant insights. For example, our own study found that cows in the RPM group had lower blood-urea nitrogen concentrations compared to the control group, along with higher levels of rumen microbial CP [91]. Consistently, academic studies have supported the positive impact of supplementing RP-Lys and RPM, combined with corn grain and soybean meal, in reducing urinary urea nitrogen excretion [92]. Further research by Ding et al. [93] confirmed the enhanced nitrogen utilization efficiency in dairy cattle through the infusion of arginine and RPM. Empirical evidence further emphasizes the beneficial effects of methionine supplementation in addressing environmental issues arising from excessive nitrogen emissions [94]. Additionally, it has been documented that a balanced combination of RP-lysine, RPM, and threonine can improve nitrogen absorption in dairy heifers, thereby contributing to reduced environmental nitrogen levels. Furthermore, concurrent supplementation of RPM and RP Lys leads to metabolic improvements and decreased nitrogen excretion [95]. These collective findings underscore the crucial role of amino acid supplementation as a promising strategy to address nitrogen-related environmental challenges and enhance nutrient utilization in livestock. Such strategies have the potential to significantly promote sustainable agricultural practices while simultaneously enhancing animal health and overall productivity.

In a study by Sobhanirad et al., the comparison of organic and inorganic zinc supplements’ effects on milk production and composition among lactating dairy cows revealed interesting insights. Although the source of zinc did not significantly affect the milk and FCM yield, the basal diet supplemented with zinc methionine (ZnM) showed potential benefits. This supplementation resulted in a higher milk and FCM yield, with a lower somatic cell count, compared to controls [96]. Consistent with these findings, an earlier study demonstrated improved lactational performance and reduced somatic cell counts in cows fed ZnM [97]. These results highlight the potential advantages of ZnM supplementation in enhancing dairy cow performance and milk quality.

### 4.2. Rumen-Protected Methionine’s Role in Ruminants’ Milk Production

Dairy animals during parturition are prone to experiencing a negative energy and protein imbalance because their nutrient intake is insufficient to meet the demands of milk production [6,8,98]. The importance of RPM in milk production has garnered significant attention in dairy cattle research [99,100,101,102,103]. Therefore, increasing milk production requires the strategic implementation of high-protein diet supplementation for dairy cattle. This approach is supported by substantial evidence, as there is a well-documented association between RP Lys, RPM, and threonine concentrations with the growth, physiology, and reproductive performance of calves [104,105]. Accordingly, Lee et al. emphasized that providing RP-Lys, RPM, and histidine in combination significantly increased milk protein yield in dairy cows fed a diet deficient in metabolizable protein [106]. Further evidence suggests that supplementing cows with 10 g/day of RPM, along with a concentrated diet containing corn grain and soybean meal, significantly increased milk production in dairy cattle [107]. This finding is consistent with the study of Carder and Weiss [108], who demonstrated that supplementation with RPM and Lys resulted in sustained increases in milk energy and milk yield. Consistently, the continuous supplementation of RPM has been linked to increased milk yield and milk protein content in periparturient dairy cows [109,110]. Studies conducted during the pre- and postpartum periods consistently showed that RPM supplementation can increase both milk yield and milk protein content in dairy cattle [13,17,111]. Giallongo et al. further supported these findings by demonstrating that combining RPM increased overall milk performance, including milk yield and milk components, in Holstein cows [33]. Furthermore, RPM supplementation has been shown to effectively enhance milk performance and address the metabolizable-protein deficiency gap in dairy cattle [106,112]. Even in the context of a diet deficient in metabolizable protein, milk production remained unaffected in dairy cattle supplemented with RPM [87]. Recent research by Park et al. reaffirms the positive impact of supplementing cows with 10 g/day of methionine, in conjunction with corn grain and soybean meal, resulting in a substantial enhancement in milk production. Additionally, studies have experimentally demonstrated the benefits of supplementing corn silage and corn milling products with RPM, yielding notable improvements in milk production among dairy cattle [113]. Furthermore, the genetic factors underlying the effects of RPM supplementation on milk production performance have been the focus of extensive investigation.

From the above discussion, it is clear that RPM supplementation plays a crucial role in addressing the challenges of negative energy and protein balance during parturition. An increasing body of research consistently demonstrates that careful RPM supplementation, along with strategic dietary adjustments, holds promise in significantly increasing milk yield and enhancing overall milk quality in dairy cattle.

### 4.3. Molecular Mechanisms Unveiling the Influence of Methionine Supplementation on Dairy Cattle Milk Production Performance

A comprehensive investigation into the genetic basis of the impact on milk production in dairy cattle due to RPM supplementation has revealed intricate molecular pathways and regulatory elements. Numerous in vitro experimental studies have diligently elucidated the regulatory functions and molecular mechanisms of cAMP response element-binding protein-regulated transcription coactivator 2 (CRTC2) in methionine-induced milk fat synthesis [45]. These studies emphasize the critical role of CRTC2 as a transcription coactivator within the methionine-induced milk fat synthesis pathway mediated by the mammalian target of rapamycin (mTOR) in BMECs.

An independent study has demonstrated that methionine supplementation has a significant influence on the expression of purine-rich element-binding protein B (PURB), a key regulator of gene transcription and cellular physiology [46]. The upregulation of PURB, in conjunction with methionine treatment, has led to increased milk protein and fat synthesis, accompanied by elevated expressions of mTOR and sterol response element-binding protein (SREBP)-1c within BMECs. Interestingly, counteractive effects were observed when PURB expression was reduced. Furthermore, an important discovery has emerged regarding the positive modulation of U2 snRNP auxiliary factor 65 kDa (U2AF65) by methionine, further enhancing milk synthesis and cell proliferation within BMECs through the mTOR-SREBP-1c signaling pathway [114].

Annexin A2 (AnxA2), renowned for its diverse roles encompassing growth, development, and metabolism, assumes a significant role in milk synthesis and cell proliferation. Methionine treatment has exhibited a positive influence on phosphatidylinositol 3-phosphate (PIP3) levels, mTOR phosphorylation, and protein levels of SREBP-1c and Cyclin D1. In this complex process, AnxA2 emerges as a critical regulator through the phosphatidylinositol-3-kinase (PI3K)-mTOR-SREBP-1c/Cyclin D1 signaling pathway [115]. Furthermore, the synergistic overexpression of glucose-regulated protein 78 (GRP78) alongside methionine treatment has yielded notable stimulatory effects on milk protein and milk fat synthesis, augmented cell proliferation, and an affirmative modulation of mTOR phosphorylation. This coalescence has also heightened protein levels of Cyclin D1 and SREBP-1c. Notably, the ablation of GRP78 through siRNA transfection has manifested contrasting outcomes. Intriguingly, the predominant cytoplasmic localization of GRP78 in bovine mammary epithelial cells has been observed. Moreover, its protein expression has been significantly enhanced following stimulation with methionine, leucine, estrogen, and prolactin [116].

Implicating the nuclear factor of κB (NFκB) family, renowned for its roles in gene expression regulation, unveils its involvement in milk synthesis regulation. NFκB1 has been identified as a governing factor for various genes, including SREBP-1c, and β4-galactosyltransferase-T2 (β4Gal-T2), consequently impacting the milk biosynthesis process [117]. Methionine treatment has been shown to enhance NFκB1’s binding to gene promoters of mTOR, SREBP-1c, and β4Gal-T2 in BMECs, illuminating milk biosynthesis to be facilitated through the PI3K pathway rather than the mTOR signaling pathway. Further insights have emerged from studies delving into the intricate regulatory network, indicating that methionine treatment leads to the suppression of DEAD-box helicase 6 (DDX6) expression. As a pivotal member of the RNA helicase family governing mRNA storage and translation regulation, decreased DDX6 expression adversely affects milk synthesis by hampering the effects of p-mTOR, SREBP-1c, and Cyclin D1 within BMECs [118]. Furthermore, gene functional analyses have corroborated the positive regulatory impact of methionine on SREBP-1c gene expression, thereby promoting milk fat synthesis through epidermal-type fatty acid binding protein-5 (*FABP-5*) [119]. Intriguingly, the heterodimeric amion acid taste receptor (TAS1R1/TAS1R3) constellation emerges as a sensor for extracellular methionine in BMECs. Activation of this receptor tandem triggers mTOR signaling, possibly via intracellular calcium-concentration elevation, and is implicated in the mediation of methionine and valine-induced changes in β-casein (CSN2) mRNA abundance [120].

A comprehensive overview of the genetic responses elicited by methionine in the regulation of milk synthesis, has been presented in Table 1. These findings collectively illuminate the intricate genetic mechanisms by which rumen-protected methionine supplementation exerts its influential role in enhancing milk production performance in dairy cattle.

### 4.4. Rumen-Protected Choline Role in Metabolism of Dairy Cattle

Choline, an indispensable nutrient, plays a central role in the field of metabolism. It is embedded in the composition of lipid-soluble metabolites, especially phosphatidylcholine, lysophosphatidylcholine, free choline and sphingomyelin, all of which are essential components of cell membranes. These compounds play critical roles in cell signaling and lipid metabolism, and their profound importance has been clearly elucidated by the scientific community [150]. The indispensable nature of choline and its metabolites underscores their important roles in maintaining structural integrity and promoting signaling functions within cell membranes. Furthermore, choline actively participates in the synthesis of acetylcholine, a highly important neurotransmitter, and also contributes to methylation processes through the efficient transfer of methyl groups, facilitated by the intermediary betaine metabolite, leading to the synthesis of the crucial SAMe pathway [151].

The prominence of choline metabolites in the biological development of mammals is particularly highlighted during the transition period. Supporting this, observations have confirmed the potential for choline supplementation during this critical phase to enhance hepatic lipid metabolism in cells, a finding supported by various studies [152,153,154]. Of note, among these choline metabolites, betaine emerges as a key facilitator in the synthesis of creatine, carnitine, and methionine. Its role as a methyl group donor, which traverses the transmethylation pathway, has been identified as instrumental in this process [155]. A spectrum of dietary sources boasts substantial phosphatidylcholine content, typically around 13% by weight [156]. Interestingly, the inclusion of choline in the diet significantly influences the remethylation activity of betaine. While the reversal of betaine into choline through a reverse reaction is not feasible, betaine assumes a supporting role under circumstances where choline availability is constrained, aiding the remethylation reaction for the synthesis of Hcy [157]. This dynamic modulation involving betaine orchestrates methylation through the Hcy methyltransferase reaction, thereby exerting regulatory control over adoHcy and S-adenosyl methionine levels within cells. This, in turn, leads to improvements in the epigenetic mechanism and DNA methylation within the cellular environment. Moreover, the presence of choline and betaine within the diet, alongside other methyl donor groups, exerts a profound influence on methylation reactions [138]. Of particular significance, the collaborative contributions of methyl-tetrahydrofolate and betaine underscore their involvement in the biosynthesis of methylation from Hcy to methionine [158].

During the pivotal transition period, dairy cows undergo significant physiological changes. These changes include a reduction in dry matter intake, an imbalance between negative energy and metabolizable protein, and an increased demand for high-quality nutrients to support both fetal growth and lactation performance. This juncture is accompanied by the emergence of potential challenges such as ketosis, hypocalcemia, clinical mastitis, and indications of fatty liver [159,160,161]. The liver functionality index (LFI), serving as a reflection of transition cow metabolic health, captures shifts in biomarkers linked to liver plasma protein synthesis (albumin), lipoprotein synthesis (cholesterol), and heme catabolism (bilirubin). Importantly, research has illuminated the connection between choline-fed cows and elevated LFI values, translating to improved dry matter intake, milk yield, milk fat yield, milk protein yield, and a reduced vulnerability to metabolic ailments [66].

During this complex transition phase, the interplay of stress and hormonal dynamics leads to an increase in NEFA in circulation, serving as a compensatory mechanism to counter the negative energy balance in transitioning cows. The liver coordinates the oxidation of NEFA to generate energy, with some of it redirected towards the production of TG, subsequently mobilized as VLDL [162]. However, it is important to note that a portion of NEFA undergoes partial oxidation in the liver, resulting in the accumulation of ketone bodies in the bloodstream, hindering the transport of triglycerides through VLDL. Additionally, in the pre- or early lactation phase for cows, there may be an elevation in the ratio of lipid peroxidation [163], a reduction in serum α-tocopherol, and increased levels of oxidative stress. This collective situation has the potential to negatively impact the health and productive performance of dairy cows [164]. Notably, RPC supplementation has been confirmed to help lower blood BHBA levels in periparturient dairy cattle [165]. Existing evidence highlights the role of RPC in improving hepatic TG levels, thereby supporting optimal production performance during the lactational period [37,166]. Introducing dietary RPC supplementation yields significant results, with a noticeable increase in colostrum production among Holstein cows [167,168]. This increase is particularly pronounced among cows in their second parity. However, it is worth considering that the demand for choline could potentially be even more pronounced among cows in their third or higher parities. Furthermore, strategic choline supplementation has been found to translate into higher concentrations of phosphocholine within colostrum. Notably, RPC supplementation has been associated with an elevation in trimethylamine N-oxide concentrations within colostrum [167,168]. A compelling study conducted by Amrutkar et al. demonstrated that RPC supplementation, administered at a dose of 54 g/day for 40 days pre-calving and 120 days post-calving, significantly increased the levels of phosphatidylcholine and vitamin E in dairy cattle [169].

### 4.5. RPC Role in Milk Production of Dairy Cattle

The pivotal role of RPC in enhancing the lactational performance of dairy cattle has been extensively studied and is concisely presented in Table 2. The use of RPC in peripartum dairy cattle has garnered significant attention due to its consistent ability to increase either milk yield or fat-corrected milk production [170]. This phenomenon has received ample confirmation in the scientific literature, with a consensus gradually emerging that the inclusion of 12-20 g per day of RPC in the diet of cows results in optimal improvements in dairy cow production performance [56,171].

An in-depth meta-analysis conducted by Arshad et al. [56] has drawn widespread attention, focusing specifically on RPC supplementation. This meticulous effort compiled data from 23 distinct experiments, encompassing 74 treatment means and a robust sample of 1,938 cows. The results derived from this meta-analysis are striking, revealing a statistically significant increase in both pre- and postpartum daily dry matter intake by 0.28 kg per day and 0.47 kg per day, respectively, with significant differences. Furthermore, this analysis unveiled a noticeable rise in ECM production, resulting in a weighted mean average increase of 1.61 kg per day. Additionally, distinct improvements were observed in fat and protein yield, with increases of 0.08 kg per day and 0.06 kg per day, respectively [56]. Consistently, Humer et al. [55] conducted an exhaustive review of data from 27 separate studies, shedding light on a significant increase in postpartum dry matter intake, from an average of 19.1 to 19.9 kg per day (*p* < 0.01), along with an increase in milk yield from an average of 31.8 to 32.9 kg per day (*p* = 0.03) following RPC supplementation. Moreover, a statistically significant increase in milk fat and protein yield was observed (*p* ≤ 0.05), while the compositional attributes of milk remained unaffected [55].

During the early stages of lactation, there is often a negative nutrient balance, leading to a decrease in choline metabolite concentrations in bovine plasma [154,172]. However, it has been discovered that RPC supplementation can reverse this trend, resulting in increased plasma concentrations of choline metabolites in prepartum cows, thereby improving their choline status [166]. Increasing choline status in the prepartum phase offers a promising approach to reduce the risk of fatty liver in cows [166,173] and positively impact postpartum health and lactation performance [174]. The research landscape in this domain involves an extensive exploration of the effects of RPC supplementation in various formulations, dosages, and dietary contexts on postpartum performance. Generally, including RPC in the diets of transition cows has yielded favorable results, demonstrating enhancements in milk, fat, and ECM yields [175,176]. However, it is essential to acknowledge the variability in responses across studies. Notably, the response to RPC supplementation has not been consistent in all investigations [121,177]. Additionally, the timing and duration of RPC feeding have been identified as pivotal factors affecting the performance of lactating Holstein cows, as elucidated by another study [38].

**Table 2:** Summary of studies associated with RPC impact on milk production and metabolism in dairy cattle.

RPC Treatment	Main Outcomes	Author
RPC at 15 g/day/head.	✓ RPC supplementation plays a substantial role as a lipotropic agent in the context of dairy ruminants. It operates by mitigating the accumulation of excess fat within hepatic cells, thereby promoting liver health. ✓ An observable outcome of RPC supplementation is a notable increase in various vital milk components. This includes a significant elevation in milk yield, as well as heightened levels of fat, lactose, solid-not-fat (SNF), total solids, and the protein yield. ✓ This cumulative effect of RPC supplementation contributes to the enhancement of milk quality, composition, and overall productivity within the dairy ruminant sector.	[171]
RPC at 54 g for 40 days before and 120 days after calving.	✓ Improved milk yield and milk component (fat, protein and lactose) yield. ✓ Suppressed the level of triglycerides and very low-density lipoproteins. ✓ Enhanced phosphatidylcholine and vitamin E levels.	[169]
Supplementing 12.9 g/d of choline ion.	✓ Supplementing 12.9 g/d of choline ions led to a 2.13 kg/d increase in ECM. ✓ This may be because choline supplementation prevents metabolic diseases and stress in lactating dairy cows.	[56]
The RPC supplementation was initiated during the final month of the transitional period and two months postparturient at 50 g/day/head.	✓ Supplementation with RPC resulted in a 2 kg/day/head increase in milk production, as indicated by the obtained results. ✓ Due to higher digestibility and increased total VFA concentration, and the prevention of metabolic disorders such as ketosis and fatty liver syndrome, this increase promotes milk fat synthesis by facilitating phospholipid synthesis, lipid absorption, and transport to the mammary gland.	[178]
The RPC product supplemented (60 g/d ReaShure, Balchem Corp., New Hampton, NY, USA) contained 28.8% choline chloride as per manufacture information, which supplied supplemented cows a daily dose of 12.9 g of choline ion.	✓ Modification of RPC increased the milk yield per animal by 2 L per day. Increased choline availability during early lactation may have stimulated the enzyme to increase mitosis in CT mammary cells. ✓ In addition, choline may exert some endocrine control over the mammary organ and influence nutrient partition toward milk synthesis via growth hormone increases. ✓ These modifications increased lipolysis and ketogenesis, which could provide nutrients for milk production. ✓ Moreover, RPC treatment decreased the liver triacylglycerol concentration of plasma in dairy cattle.	[38]
RPC supplementation.	✓ Milk production enhanced, decreased liver triacylglycerol concentration of plasma.	[179]
RPC supplementation.	✓ Increased yields of milk by 1.8 kg/d, fat by 0.08 kg/d, lactose by 0.08 kg/d, true protein by 0.04 kg/d, ECM by 1.9 kg/d, and fat-corrected milk by 2.1 kg/d without dry matter intake being affected.	[37]
200 g RPC after calving for 60 days of lactation, which provided 50 g per day choline.	✓ Throughout the research, RPC supplementation had a significant impact on milk production. During the first 60 days of lactation, the milk yield increased by 4.41 kg/day, and as a result, 4% FCM increased by 2.5 kg/day on average.	[180]
22 g choline ion from an established product (prepartum: 0.10 ± 0.004 choline ion, %DM.	✓ In general, peripartum RPC supplementation at the suggested dose tended to increase ECM yield after the supplementation period. ✓ This is because the effects of RPC on metabolic and inflammatory biomarkers support the potential for RPC supplementation to influence the metabolism and health of transition cows and may contribute to the observed production gains.	[181]
RPC at 15 to 50 g/day/head.	✓ The RPC effect increased milk yield by an average of 1.05 kg per day. Additionally, the yields of milk protein and milk lipids were increased. This may be due to the prevention of metabolic condition, stress and dry matter intake. ✓ The RPC intake improved energy and nutrient intake is anticipated to have contributed to the reduction in NEB in early-lactating cows. This ameliorating effect is anticipated to manifest as either an enhanced NEB, i.e., less fat mobilization, reflected in less BW loss, lower circulating NEFA and BHBA, and higher glucose and cholesterol, or increased milk yields.	[55]
The RPC supplemented at 40 g/d/head.	✓ Increasing the intestinal supply of choline improved milk production in lactating dairy cows by approximately 7% over controls. ✓ Supplementation of RPC increased the milk production for the following reasons: higher digestibility and increased total VFA concentration, decreased NH3-N, and the prevention of metabolic disorders such as ketosis and fatty liver syndrome.	[35]
RPC at 54 g for 90 days (pre- and postcalving).	✓ In comparison to the control group, the prill fat alone- and prill fat + rumen-protected choline-supplemented groups produced 12.10 and 21.76% more milk (kg/d) on average. ✓ The higher milk production in supplemented groups was attributed to higher TDN, which increased the energy density of the ration and reduced the deleterious effect of a negative energy balance, as evidenced by lower blood NEFA levels.	[182]
RPC at 10 g 20 days pre-calving and 64 days post-calving.	✓ RPC supplementation increased milk yield up to 7.87% higher, compared to the without-RPC group during 21–64 DIM. ✓ This is due to the fact RPC is used for liver protection, enhances fat metabolism in the liver, and reduces triglyceride levels simultaneously. As a consequence, the liver generates more glucose, which is utilized for increased milk synthesis.	[183]
RPC at 30 g for 8 weeks after calving.	✓ The incorporation of RPC into the diet yielded noteworthy improvements in actual milk production, alongside a 4% increase in the yield of FCM. ✓ When compared to the unsupplemented RPC regimen, the addition of 30 g of RPC led to a substantial rise in actual milk yield, amounting to 2.24 kg per head per day, reflecting a remarkable increase of 14.85%. ✓ Improved nutrient digestibility and elevated concentrations of total volatile fatty acids (TVFA), coupled with reduced ammonia–nitrogen (NH3-N) concentration within the rumen of animals receiving RPC supplementation. ✓ RPC supplementation was associated with enhanced milk yield in high-producing dairy cows during the initial 60 days of lactation, potentially by augmenting lipid export from liver metabolism. ✓ It is recommended to employ RPC supplementation for high-yielding dairy herds, particularly in the peri-parturient period and especially when cows exhibit excessive body condition. ✓ Feeding RPC induces an increase in choline concentration and milk yield, indicative of augmented choline availability among supplemented cows. ✓ The substantial elevation in milk production is a direct outcome of metabolic adaptations induced by RPC supplementation, underlining the multifaceted impact of RPC on dairy cow physiology and milk performance.	[184]
RPC and vitamin B supplementation.	✓ Decreased liver fat and enhanced milk production.	[185]
60 g/d of RPC (13.0 g/d of choline ion).	✓ RPC increased the overall milk yield of primiparous cows by 3.1 kg/day. This may be due to a decrease in the negative energy balance and metabolic diseases due to choline supplementation.	[186]
Supplemented 60 g of RPC/d (providing 15 g choline chloride) during the first 10 weeks of lactation.	✓ In the initial 10 weeks of lactation, dairy cows fed a total mixed ration (TMR) experienced enhanced milk production outcomes through the supplementation of 60 g of RPC (rumen-protected choline) per day. ✓ This supplementation regimen provided 15 g of choline chloride to the cows’ diet. ✓ As a result of this supplementation strategy, the yield of FCM with a fat content of 4% (4% FCM) increased significantly by 3.28 kg per day. ✓ Additionally, there were notable improvements in the yields of both fat and protein from the milk produced by the supplemented cows.	[187]
RPC at 30 g/day per cow for the first 16 d of lactation.	✓ Cows supplemented with RPC at a rate of 30 g per head per day exhibited an increase in milk production of 3.8 kg per day, as compared to control cows, within the initial 16 days of lactation. ✓ RPC supplementation might have contributed to the reinforcement of the cows’ antioxidant defense mechanisms, resulting in a reduction in oxidative stress within their systems. ✓ This supplementation regimen could have led to an elevation in the concentrations of essential nutrients such as vitamin E and methionine in the cows’ physiology. ✓ Vitamin E, known for its potent antioxidant properties, could have played a role in minimizing oxidative stress, promoting better overall health, and potentially leading to increased milk production. ✓ Increased methionine concentrations, facilitated by RPC supplementation, could have positively influenced protein synthesis and metabolic processes associated with milk production.	[188]
RPC supplementation.	✓ Enhanced milk production and improved metabolism.	[189]
RPC was supplemented at 17.3 g/d/per cow from 21 days before the calving date to 21 days after.	✓ The ingestion of RPC exhibited a propensity to elevate milk production by approximately 2.2 kg/d per cow, even in the absence of discernible enhancements in liver function. ✓ This notable enhancement in milk production could potentially be attributed to amplified DMI and heightened energy mobilization. ✓ Cows subjected to RPC demonstrated a heightened intake of feed dry matter by 0.6 kg/d, indicating a potential avenue for the observed milk production increase. ✓ Cows consuming RPC showcased the elevated oxidation of fatty acids for energy during the initial stages of lactation. This energy diversion potentially conserved glucose for augmented lactose synthesis, thereby contributing to increased milk production.	[176]
RPC at 60 g/d.	✓ RPC is postulated to hold a higher likelihood of conferring favorable outcomes in overfed calves, as their susceptibility to hepatic triglycerides accumulation is elevated. ✓ The introduction of supplemental RPC resulted in heightened milk production among cows possessing a BCS of 4 at the commencement of the lactation phase. This increase is predominantly attributed to the augmented DMI. ✓ Based on these empirical findings, a hypothesis was formulated suggesting that supplementing herds where more than 20% of cows entered the transition period with a BCS of 4, with RPC, could potentially yield a positive impact on milk production.	[15,121]
Dose of RPC was from 15 to 50 g.	✓ RPC supplementation exhibited a significant enhancement in milk yield during the commencement of lactation. ✓ RPC serves as both a precursor for acetylcholine and a vital source of methyl groups, facilitating the remethylation of Hcy into methionine. ✓ RPC supplementation potentially exerts a sparing influence on methionine, a pivotal amino acid in milk protein synthesis for lactating dairy cows, possibly alleviating its constraint. ✓ RPC’s capacity to conserve methionine by providing methyl groups emerges as a conceivable mechanism, potentially contributing to the heightened milk protein yield. ✓ RPC supplementation led to an increase in DMI, consequently bolstering the availability of energy and nutrients at the onset of lactation. ✓ The observed surge in milk yield could also be attributed to the elevated energy and nutrient supply facilitated by increased DMI.	[190]
RPC	✓ Improved feed efficiency (FE) and average daily gain (ADG) and increased growth. ✓ Enhanced plasma glucose level. ✓ Elevated serum insulin-like growth factor-1 and suppressed expression of serum LPS-binding protein.	[191]
RPC	✓ Enhanced the biosynthesis of milk protein via the dephosphorylation of EEF2 and EIF2A during the negative nutrient balance in periparturient dairy cattle.	[192]
RPC	✓ Regulates the liver metabolism of periparturient dairy cows via activation of metabolic processes such as fatty acid synthesis and metabolism and glucose metabolism.	[193]

## 5. Conclusions

Altogether, addressing the challenges posed by nitrogen pollution while simultaneously enhancing milk production performance in dairy animals has become a crucial concern for sustainable livestock production. Another key concern is negative energy balance, which compromises the metabolism and health and consequent lactational performance of periparturient dairy cattle. This review article examined the significant roles of RPM and RPC in optimizing metabolism, nitrogen utilization, and improving milk production performance in dairy cattle. These strategies have emerged as powerful tools to mitigate the environmental impact of excessive nitrogen excretion while boosting the overall productivity of dairy animals. The incorporation of RPM into dairy cattle diet has been shown to have multifaceted benefits, ranging from its role as a limiting amino acid for milk production to its involvement in sulfur-containing amino acid synthesis and key metabolic pathways. Research has consistently demonstrated that their supplementation positively influences milk yield, milk protein synthesis, and overall metabolic efficiency. Similarly, RPC has been found to play a pivotal role in supporting metabolic processes during the transition period and beyond, contributing to improved liver health, lipid metabolism, and overall milk production performance.

## Figures and Tables

**Table 1:** Summary of studies investigating the influence of RPM on metabolism and milk production performance of dairy cattle.

Amino Acid Supplementation	Main Outcomes	Species	Author
RPM and RPC	✓ Enhanced metabolism and milk production performance	Dairy cattle	[121]
RPM	✓ Improved metabolism, enhanced milk production	Dairy cattle	[122]
RPM	✓ The RPM supplementation promoted higher milk yield, ECM yield, milk protein yield, milk protein, casein and milk fat yield	Dairy cattle	[123]
RPM	✓ Increased milk yield and milk components yields and metabolism	Dairy cattle	[112]
ZnM	✓ Improved lactational performance ✓ Facilitated metabolic processes, enhanced nitrogen utilization, and decreased environmental pollution	Dairy cattle	[98,124]
RPM and RPC	✓ Enhanced milk production performance, increased postpartum dry DMI, regulated hepatic lipid metabolism, and improved immunity	Dairy cattle	[125]
Hydroxyselenomethionine	✓ Enhanced lactation performance, and milk Se concentrations in early-lactating dairy cows	Dairy cattle	[126,127]
*N*-acetyl-l-methionine supplementation (NALM)	✓ Enhanced milk production ✓ Increased concentrations of total protein and globulin in plasma ✓ Improved nitrogen absorption and alternative reduction in environmental pollution	Dairy cattle	[128,129]
NALM s	✓ Facilitated metabolism with improvement in milk production performance	Dairy cattle	[130,131]
RPM	✓ Enhanced metabolism of carnitine and enhanced β-oxidation of fatty acids, and improved cholesterol metabolism followed by Lipoprotein metabolism ✓ Promoted one-carbon-metabolism cystathionine and beta-synthase-activity cystathionine, followed by enhancement of antioxidant synthesis	Dairy cattle	[132]
RPM	✓ Enhanced digestion and milk production ✓ Improved utilization of nitrogen absorption	Dairy cattle	[133]
RPM and RPC	✓ Enhanced metabolism and milk production, and decreased incidence of metabolic issue	Dairy cattle	[134]
16% CP and 25 g/head/day RPM	✓ Enhanced milk production phenotypes (milk casein, milk yield and milk fat yield)	Dairy cattle	[135]
RPM and RP Lys	✓ Promoted yield of milk, fat, protein, and lactose ✓ RPM and RPL did not change milk production phenotypic traits in adequate amino acids diet	Dairy cattle	[136]
RPM+RP Lys	✓ Milk production performance including milk yield and the content of fat and protein were significantly enhanced ✓ Metabolism was improved and it prevented body weight loss	Dairy cattle	[137]
RPM+fava bean	✓ Improved DMI followed by enhancement of milk protein yield ✓ Nitrogen-use efficiency for milk production tended to increase by decreasing nitrogen excretion ✓ Elevated plasma methionine	Dairy cattle	[138]
RPM and RP Lys for 21 days	✓ Increased DMI and increased milk yields, and yield of milk protein, solids, lactose, and milk lactose concentration ✓ Promoted milk Se ng/mL, milk N g/kg and milk Se:N, ng/kg	Dairy cattle	[139]
RPM	✓ Significantly enhanced the milk yield, milk protein percentages and milk fat percentages	Dairy cattle	[140]
RPM and RPC	✓ Enhanced metabolism and amino acid profile in plasma followed by improved lactational performances	Dairy cattle	[121]
RPM	✓ Induced ARID1A degradation to promote mTOR expression and milk synthesis in mammary epithelial cells	BMECs	[41]
RPM	✓ Increased the expression of PURB to stimulate mTOR and SREBP-1c, followed by increased milk protein and fat synthesis	BMECs	[46]
RPM	✓ Enhanced the expression of Twinfilin1 (TWF1) which, by using the mTOR pathway, enhances the milk bio-synthesis and cell proliferation	BMECs	[141]
Methionyl-methionine dipeptide	✓ Regulated the synthesis of β-CSN in BMECs. ✓ Increased the mRNA abundance of Janus kinase 2 (JAK2) and signal transducer and activator of transcription 5 (STAT5), leading to the enhanced phosphorylation of JAK2, STAT5, mTOR, p70 ribosomal S6 kinase 1, and eukaryotic initiation factor 4E binding protein 1. ✓ Inhibition of both JAK2 and mTOR significantly reduced the Met-Met-induced increase in cell viability and β-CSN synthesis in BMECs.	BMECs	[142,143]
RPM	✓ β-CSN expression was enhanced followed increased level of mTOR and SREBP-1c, resulting in milk and milk fat synthesis	BMECs	[144]
RPM	✓ Biosynthesis of milk β-CSN via regulating mTORC2-protein kinase B (AKT) signaling in methionine (Met)-induced L-type amino acid transporter 1 (LAT1) ✓ Upregulated milk protein synthesis	BMECs	[145]
RPM	✓ Activates the TAS1R1/TAS1R3 receptor, which appears to function as a sensor for extracellular methionine in bovine mammary cells, leading to the activation of mTOR signaling, possibly through the elevation of intracellular calcium concentration and mediating the changes in CSN2 mRNA abundance ✓ Also promoted the activation of CSN1S1 and eukaryotic elongation factor (eEF) 2 to enhance milk biosynthesis	BMECs	[120,146,147,148]
RPM	✓ Enhanced mTOR phosphorylation via Wnt-induced secreted protein 3 (WISP3), which is followed by improved milk fat synthesis	BMECs	[149]
